# A Robust Strategy for Negative Selection of Cre-LoxP Recombination-Based Excision of Transgenes in Induced Pluripotent Stem Cells

**DOI:** 10.1371/journal.pone.0064342

**Published:** 2013-05-22

**Authors:** Syandan Chakraborty, Nicolas Christoforou, Ali Fattahi, Roland W. Herzog, Kam W. Leong

**Affiliations:** 1 Department of Biomedical Engineering, Duke University, Durham, North Carolina, United States of America; 2 Department of Pharmaceutics, Faculty of Pharmacy, Kermanshah University of Medical Sciences, Kermanshah, Iran; 3 Department of Pediatrics, University of Florida, Gainesville, Florida, United States of America; Johns Hopkins University, United States of America

## Abstract

Viral vectors remain the most efficient and popular in deriving induced pluripotent stem cells (iPSCs). For translation, it is important to silence or remove the reprogramming factors after induction of pluripotency. In this study, we design an excisable loxP-flanked lentiviral construct that a) includes all the reprogramming elements in a single lentiviral vector expressed by a strong EF-1α promoter; b) enables easy determination of lentiviral titer; c) enables transgene removal and cell enrichment using LoxP-site-specific Cre-recombinase excision and Herpes Simplex Virus-thymidine kinase/ganciclovir (HSV-tk/gan) negative selection; and d) allows for transgene excision in a colony format. A reprogramming efficiency comparable to that reported in the literature without boosting molecules can be consistently obtained. To further demonstrate the utility of this Cre-loxP/HSV-tk/gan strategy, we incorporate a non-viral therapeutic transgene (human blood coagulation Factor IX) in the iPSCs, whose expression can be controlled by a temporal pulse of Cre recombinase. The robustness of this platform enables the implementation of an efficacious and cost-effective protocol for iPSC generation and their subsequent transgenesis for downstream studies.

## Introduction

Reprogramming of somatic cells into a pluripotent-stem-cell-like state by retrovirus-based expression of Yamanaka transcription factors (Oct3/4, Sox2, Klf4 and c-Myc) have opened up new vistas in using lineage-specific cells for disease modeling, drug screening, developmental biology studies and cell-based therapies [1]–[11]. Since then cellular reprogramming has been achieved by the delivery of transcription factors via lentivirus, adenovirus, plasmid, transposon, mRNA or protein [12]–[17]. Out of these methods the lentiviral approach is preferred because of its high efficiency of transgene insertion and pluripotency induction, except for translation [18]. The integrative nature of lentiviral transgenesis would require excision of the transgenes after reprogramming. It has been demonstrated that iPSCs with their transgenes excised resemble ES cell-lines more than the iPS cell-lines that have the reprogramming transgenes unexcised [19]. Moreover, expression of the reprogramming factors may be aberrantly activated in the iPSCs to interfere with differentiation. Therefore, investigators have looked at various ways to remove the transgenes after lentiviral reprogramming.

Cre recombinase-mediated excision of LoxP-flanked reprogramming cassette has been extensively reported in the literature. This approach is usually based, firstly, on a transient expression of a GFP-tagged-Cre expression cassette in the reprogrammed cells. Subsequently, GFP+ cells are sorted and screened for transgene excision [20]. This procedure assumes that almost all the GFP+ cells will have their transgenes removed by Cre recombinase. However, due to the frequent cell division in iPSCs leading to quick dilution of the Cre recombinase transcripts and the stochastic nature of Cre recombinase-mediated excision [21], it is unlikely that most of the iPSCs will have their reprogramming transgenes excised. This method therefore mandates an extensive and laborious screening procedure for isolating the iPSCs with excised transgene. Secondly, a florescent-protein tag is included in addition to the reprogramming factors in the construct to facilitate sorting [22]. This was done by replacing the cMyc gene with mCherry (a fluorescent protein) due to space constraint in a lentiviral vector. However, exclusion of c-Myc led to a loss of reprogramming efficiency and hindered maturation of the differentiated lineages [23]. Both the aforementioned approaches require fluorescence-activated cell sorting (FACS) that apart from being costly, is not often easily accessible to the investigators. Moreover, these methods necessitate the disruption of a colony to make a single cell suspension for FACS. This step, especially for human iPSC derivation, is undesirable because pluripotent cells in isolation experience selection pressure and hence are prone to growth-promoting chromosomal aberrations [24].

After reprogramming, often a need arises to further engineer the iPSCs for downstream applications. This may be transgenes to tag stem cells for lineage tracking [25], [26] or to express genes for therapeutic and developmental studies [27]–[30]. Antibiotic selection is often used for the selection of transgene-integrated pluripotent stem cells. However, continued antibiotic expression can affect the expression of the transgene of interest. Many protocols have been reported to remove the selection cassette by using site-specific recombinase technology [31]–[33]. However these techniques face the same drawbacks mentioned earlier. In addition to the ability to express a transgene for downstream studies it is often necessary to have a temporo-spatial control over the expression. It can be achieved by expressing Cre recombinase under the control of a tissue specific promoter [34], [35] and/or by a transient temporal pulse of Cre recombinase. Therefore, these considerations (post-transgenesis removal of selection cassette and temporo-spatial control) have prompted us to develop a vector-based solution by utilizing Cre-loxP recombinase technology with subsequent HSV-tk/ganciclovir-based negative selection.

In this study, we design an excisable loxP-flanked lentiviral construct that a) includes all the reprogramming elements in a single lentiviral vector expressed by a strong EF-1α promoter; b) enables easy determination of lentiviral titer; c) enables transgene *removal* and cell enrichment using LoxP-site-specific Cre-recombinase excision and Herpes Simplex Virus-thymidine kinase/ganciclovir (HSV-tk/gan) negative selection; and d) allows for transgene excision in a colony format. To further demonstrate the utility of this Cre-loxP/HSV-tk/gan strategy in downstream application of iPSCs, we also show the incorporation of a non-viral therapeutic transgene (human blood coagulation Factor IX) in the iPSCs, whose expression can be controlled by a temporal pulse of Cre recombinase.

## Methods

### Vector design and construction

PlasmaDNA software (University of Helsinki) was utilized for the *in silico* cloning. Self-cleaving 2A-peptide-linked reprogramming factors: Myc, Klf-4, Oct-4, Sox-2 (MKOS) were cloned from the Addgene Plasmid 20866: pCAG2LMKOSimO. The MKOS fragment was cut out with EcoRI (NEB-New England Biolabs) and was subsequently blunted with DNA Polymerase I, Large (Klenow) Fragment (NEB). pWPXL (Addgene Plasmid 12257) vector was linearized with SpeI (NEB) and BamHI (NEB) which resulted in the removal of the GFP (Green fluorescence protein) fragment. The vector was blunted with DNA Polymerase I, Large (Klenow) Fragment. The fragments were then ligated with T4 DNA ligase (NEB). The ligation resulted in a plasmid which was labeled as pMKOS-WPXL. In pMKOS-WPXL the MKOS fragment was expressed by the strong EF1alpha promoter of pWPXL. In the next cloning step IRES (internal ribosome entry site)-HSV-tk (Herpes Simplex Virus-Thymidine Kinase) was PCR amplified from Addgene Plasmid 12243: pLOX-gfp-iresTK by utilizing the primers tcgagctcaagcttcgaatta and taaaggtaccgtcgagccaaa. Phusion high-fidelity polymerase (Finnzymes) was used for the amplification. The cycling parameters were: Initial denaturation: 98°C×30 sec; Denaturation: 98°C×5 sec, Extension: 72°C×60 sec, for 35 cycles; Final extension: 72°C×10 min; Hold: 4°C. The PCR product was phosphorylated with T4 Polynucleotide Kinase (NEB). It was then ligated to pMKOS-WPXL that was linearized with NdeI (NEB) and blunted with Klenow enzyme. The final form had a MKOS and a HSV-tk cassette with an internal ribosome entry site (IRES) in between them; both driven by a strong EF1 alpha promoter. The plasmid was named as pMKOS-TK-WPXL.

For demonstrating the utility of Cre-lox/HSV-tk/Gan technology in achieving removal of selection cassette and temporo-spatial control over transgene expression, the vector pCag-loxP-PGK-HSV-tk- BlastR-tpA-loxP-FIX was constructed. Gateway recombination cloning technology (Life technologies, Grand Island, NY) was utilized to generate the vector. The blood coagulation factor IX Gateway entry vector (pENTRY4-FIX) was constructed by inserting a codon optimized version of human factor IX cDNA (GeneArt, Regensburg, Germany) in the gateway entry vector pENTRY4. The Gateway destination vector: pCag-loxP-PGK-HSV-tk-BlastR-tpA-loxP-Dest was constructed by utilizing multiple steps of cloning.

#### Step 1

A CAG promoter (cytomegalovirus early enhancer element and chicken beta-actin promoter) was inserted into the destination vector pRosa26-DEST (Plasmid 21189 Addgene) just proximal to the 1^st^ loxP site. It was achieved by ligating the 3.2 kbps EcoRV-AscI fragment from pRosa26-DEST with the 11.7 kbps AscI-NruI fragment from plasmid Ai9 (Addgene Plasmid 22799).

#### Step 2

A HSV-tk cassette (cut out with SalI and EcoRI from Invivogen plasmid pORF-HSV-tk) was inserted proximal to the IRES site in pVitro1-Blast (Invivogen) by utilizing the BamHI restriction endonuclease cut site.

#### Step 3

HSV-tk IRES BlastR cassette was cut out from the plasmid from step 2 (NcoI and EcoRI) to be cloned distal to the PGK (phosphoglycerate kinase) promoter in PGKdtabpA (Addgene plasmid 13440) by replacing the dta (diphtheria toxin) cassette (mobilized with restriction endonucleases NcoI and BclI).

#### Step 4

PGK-HSV-tk IRES BlastR cassette from the plasmid in step 3 (mobilized with NotI and EcoRI) was inserted into the plasmid PGKneotpAlox2 (Addgene plasmid 13444) by replacing the PGK neomycin resistance cassette (removed by HindIII and EcoRI digestion). This step allowed for the introduction of a triple polyA STOP cassette (tpA) distal to the selection cassette.

#### Step 5

LoxP-PGK-HSV-tk-IRES-BlastR-tpA-loxP cassette from step 4 was exchanged with the loxP flanked area of the plasmid from step 1 by *in vitro* homology-based Cre-mediated cassette exchange to form the gateway competent destination vector pCag-lox-PGK-HSV-tk-BlastR-tpA-lox-Dest.

In the final step of cloning the destination vector in step 5 (pCag-lox-PGK-HSV-tk- BlastR-tpA-lox-Dest) was recombined with the FIX gateway entry vector (pENTRY4-FIX) by LR recombinase (life technologies) to form the final expression vector pCag-loxP-PGK-HSV-tk-BlastR-tpA-loxP-FIX (Figure S1).

### Lentivirus production, concentration and titration

Lentiviral particles were produced by transfecting HEK 293T cells with the plasmid pMKOS-TK-WPXL along with 2^nd^ generation packaging plasmids (Addgene plasmid 12260: psPAX2 and Addgene plasmid 12259: pMD2.G). One million HEK-293T cells were seeded on a 75 cm^2^ surface area tissue culture flask. The HEK-293Ts were cultured with 10% fetal bovine serum (FBS) (Atlanta Biologicals) in DMEM-HG (GIBCO-11960) supplemented with L-glutamine, pyruvate and MEM-NEAA (GIBCO). Calcium chloride particle-based transfection method was utilized to deliver the plasmids once the HEKs were 70% confluent. 12.5 ml of fresh medium was added to the culture two hours before the transfection. The plasmid particles for transfection were produced by mixing 14 µg of MKOS-TK, 8 µg of PSPAX2, 4.3 µg of pMD2.G, 363 µl of TE 0.1X, 192 µl of water and 62.15 µl of CaCl2 2.5M. Then 627 µl of HBS2x was added to the mixture drop by drop under continuous vortexing. After 16 hours of incubation of the HEKs with the particles the medium was changed to fresh 10 ml of media supplemented with 4 mM (final concentration) of caffeine. Media was replaced three more times with an interval of 12 hours between the changes. The last media change was done without caffeine. 40 ml of the supernatant was collected and centrifuged at 250 g for 5 min to pallet out the cellular debris. The supernatant was then filtered with a .45 µm syringe filter. The filtered supernatant was concentrated up to 50× with Amicon Ultra 100 kDa filter (Millipore, cat. code UFC910008). The centrifugation was done at 2500G. The concentrated virus was then stored at −80°C for future use.

For determining the titer of the lentivirus, 25,000 primary mouse embryonic fibroblasts (PMEFs) were seeded in each well of a 6 well plate (BD falcon). Increasing quantities of the concentrated viral supernatant: 0 µl, 2 µl, 10 µl, 25 µl, 50 µl, 100 µl, were added to each well. Sequebrene was also added to the medium at a concentration of 8 µg/ml. Medium was replaced after 2 days with Ganciclovir added to the medium at a concentration of 4 µM. Ganciclovir-supplemented media was replaced every 2 days. The cell numbers were assessed after 5 days of Ganciclovir selection under a phase contrast microscope. After 7 days of selection the cells were fixed and stained with DAPI and observed under a fluorescence microscope.

### Cell culture and iPSC production

Reprogramming was induced in mouse embryonic fibroblasts (MEFs) derived from a Myh-6 GFP transgenic mouse (alpha-Myosin heavy chain promoter driven green fluorescence protein). Transgenic MEFs isolated from mouse embryos (13 d.p.c.) were expanded in standard growth media conditions [36]. For the isolation of primary mouse embryonic fibroblasts, a single pregnant female mouse was euthanized by CO2 asphyxiation. Animal procedures were conducted according to the guidelines for the care and use of laboratory animals set and approved by the Institutional Animal Care & Use Committee (IACUC) of Duke University & Duke University Medical Center. Passage 2 MEFs were seeded at a density of 25,000/well of a 6-well plate coated with .1% gelatin. After 16 hours of culture the cells were transduced with 100 ul of concentrated and titrated MKOS-TK lentivirus. 24 hours later the transduced cells were passaged to a 10 cm dish coated with 0.1% gelatin. The medium was changed to iPSC medium 2 days after the passaging. iPSC media was produced by conditioning 10% FBS in DMEM-HG supplemented with L-glutamine, sodium pyruvate and MEM-NEAA (GIBCO) with STO-SNL cell line (Soriano ES Feeder cell line SNL 76/7 STO) for 24 hours. The STO-SNL cell line was purchased from Mutant Mouse Regional Resource Center (MMRRC; Catalogue # 015892-UCD-CELL). The conditioned medium was mixed with fresh 20% FBS in DMEM-HG medium at a ratio of 1∶1. The final FBS concentration of the conditioned medium was 15%. By the 10^th^ day of reprogramming well defined colonies started appearing. On day 14 individual iPSC colonies were picked up and passaged on Mitomycin-C inactivated feeder layer of MEFs.

### Reprogramming cassette excision and verification

The excision of the MKOS-IRES-TK cassette was achieved by the transient transfection of a plasmid expressing a fusion protein of Cre recombinase and GFP (Addgene plasmid 13776: pCAG-Cre:GFP). Individual iPSC clones were passaged on a feeder layer of Mitomycin-C-inactivated PMEF (Millipore) in a 24-well TCPS plate. pCAG-Cre:GFP transfection was done by Stemfect MESC2 transfection reagent (Stemgent) following the manufacturers protocol. The iPSCs were cultured for the next 5 days with daily media change so as to allow for the excision of the MKOS-TK cassette. On the 6^th^ day Ganciclovir (Invivogen) selection was started at a final concentration of 4 µM. The excision of the construct in the surviving iPSC cells were probed by genomic PCR. Two sets of primers were used to probe the presence/absence of the construct. Genomic Gapdh primers were used as a control for the PCR. Primer set 1 extended from Klf-4 to 2A (CAGGCGAGAAACCTTACCAC, AGACTTCCTCTGCCCTCTCC- 202) and primer set 2 amplified a region extending from WPRE to HSV-TK (GGAGGATTGGGGACAGCTT, CATAGCGTAAAAGGAGCAACA-466). The genomic DNA was extracted by DNAeasy Blood and Tissue kit (Qiagen). Genomic PCR was done with the terra PCR kit from Clontech. The cycling conditions were: 98°C for 2 min; 98°C for 10 sec, 60°C for 15 sec, 68°C for 40 sec, repeated for 40 cycles.

### iPSC characterization

- iPSC differentiation: It was done by implementing the protocols for the differentiation of pluripotent cells into ectoderm, mesoderm and endoderm. For neuroectoderm differentiation, the iPSCs were dissociated with .05% trypsin-EDTA solution (GIBCO). iPS cells were then plated on 0.1% gelatin-coated surface of a 12-well TCPS plate at a density of 5×10^4^ cells/well. N2B27 medium was used for inducing the differentiation. The medium was changed every 2–3 days. For cardiac mesoderm differentiation the cells were cultured in a hanging droplet format for 2 days. Each droplet contained 500 cells in a 25 µl volume. The medium composed of 20% FBS in DMEM (High glucose, GIBCO) supplemented with L-glutamine, sodium pyruvate and MEM-NEAA (GIBCO). To promote cardiac differentiation L-Ascorbic acid (Sigma) was added at concentration of 10 ug/ml. On the 3^rd^ day of differentiation the embryoid bodies were transferred to a 0.1% gelatin-coated surface. Medium was changed every day. For the endoderm differentiation the dissociated iPS cells were suspended in a medium which consisted of 15% fetal bovine serum in DMEM, supplemented with 300 µM Mono Thioglycerol (Sigma) and 2 mM L-Glutamine (GIBCO). The cells were cultured as hanging droplets for 2 days at a density of 300cells/30 ul of media. After 2 days of droplet culture, cells were transferred to a gelatin-coated surface.
- Staining of iPSCs and their differentiated derivatives: Cells were fixed with 4% paraformaldehyde for 15 minutes. The fixed cells were permeabilized with 0.2% Triton X-100. IPSCs were then stained with primary antibodies against OCT4 (Rabbit polyclonal, ABCAM: ab19857), SSEA-1 (Mouse monoclonal, Developmental Studies Hybridoma Bank, MC-480) and NANOG (Rabbit polyclonal, ABCAM: ab80892) at a dilution of 1∶100 for 2 hours and subsequently were incubated with fluorophore-labeled secondary antibodies (Life technologies) at a final dilution of 1∶500. The differentiated cells were stained with TUJ1 (Mouse monoclonal, Stemgent: 09-0076), alpha-ACTININ (ABCAM, ab-9465), AFP (Goat polyclonal, Santa Cruz Biotechnologies: sc-8108) antibodies. Alexa Fluor 546 Goat Anti-mouse IgM was used as the secondary antibody for SSEA-1 ICC (Life technologies, A-21045). Alexa Fluor 488 Donkey Anti-goat IgG was used as the secondary antibody for AFP ICC (Life technologies, A-11055). For the other markers, either Alexa Fluor 488 Goat Anti-mouse IgG (Life technologies, A-11001) or Alexa Fluor 488 Goat Anti-rabbit IgG (Life technologies, A-11008) was used as the secondary antibody for ICC. Alkaline phosphatase staining was done with the Stemgent alkaline phosphatase staining kit II according to manufacturer's protocol.
- RT-PCR of iPSCs and their differentiated derivatives: Total RNA was extracted with a Qiagen RNeasy mini kit. RT-PCR was done by utilizing Qiagen One Step RT-PCR kit. The cycling conditions were: Reverse transcription: 50°C for 30 min; Initial PCR activation step: 95°C for 15 min; Denaturation: 94°C for 30 sec, Annealing: 58°C for 30 sec, Extension: 72°C for 60 sec, for varying number of cycles; Final Extension: 72°C for 10 min. The primers utilized for the reactions are referred to in the manuscript in a tabulated form as Table S1.

### Generation of human blood coagulation factor IX secreting iPSCs

The iPSCs were transfected with the pCag-loxP-PGK-HSV-tk-BlastR-tpA-loxP-FIX construct linearized with PacI and AscI (NEB). 10 ug of the construct was electroporated into 1 million iPSCs by using the AMAXA nucleofaction protocol A023 (Lonza). Selection of the successfully transfected cells were done by exposing the cells to 10 µg/ml of Blasticidin (Invivogen) for 4 days. Subsequently removal of the HSV-tk IRES Blast cassette was achieved by the transient transfection of CRE:GFP. Mouse anti-human FIX primary antibody (hematologic technologies, AHIX-5041) at a concentration of 20 ug/ml was used for immunocytochemistry. Alexa Fluor 488 Goat Anti-Mouse IgG (Life technologies, A-11001) was used as the secondary antibody. Forward primer: TCCATCGTGAACGAGAAGTG and reverse primer: TAGTTGTGGTGGGGGATGAT was used to detect FIX by RT-PCR. FIX chromogenic assay (Aniara, Biophen) was used to perform the functional test. 50,000 iPS cells seeded for 48 hours in a single well of a 6-well plate was used to condition the media for the chromogenic assay. 2 ml of 6 µg/ml of Vit.K-supplemented N2B27 medium was applied to the cells for 24 hours to condition the media. The conditioned media was then used for the chromogenic assay following the manufacturer's protocol.

## Results

**Figure 1:**
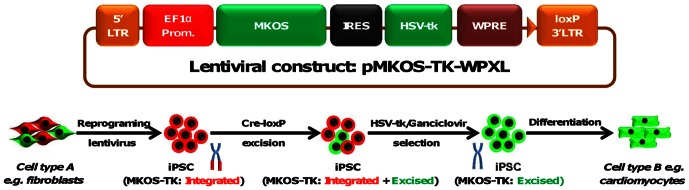
Schematic diagram for the generation of reprogramming-transgene-free iPSCs based on Cre-loxP/HSV-tk/gan selection strategy. pMKOS-TK-WPXL lentiviral reprogramming construct includes a strong EF1-alpha (EF1-α) promoter driving the MKOS (Myc, Klf-4, Oct-4, Sox-2) and HSV-tk genes. The post-transcriptional regulatory element of *woodchuck* hepatitis virus (WPRE) ensures high level of transgene expression. The construct has a LoxP at the 3′ long terminal repeat (LTR) which gets copied during lentiviral reverse transcription to the 5′LTR thereby flanking the transgene. The lentiviral particles generated from the construct can be used to transduce committed cells of any lineage (*Cell type A e.g. Fibroblasts*) and to reprogram into a primitive pluripotent cell type (iPSC MKOS-TK-Integrated). The iPSCs bearing the construct is then subjected to a transient pulse of Cre recombinase followed by Ganciclovir (a prodrug for HSV-tk-based negative selection) exposure to ensure the successful excision of the transgene. These transgene-free iPSCs (iPSC MKOS-TK-Excised) can then be differentiated to committed cells of any lineage (*Cell type B e.g. cardiomyocytes*).

The lentiviral reprogramming construct had a strong EF-1alpha promoter which is known to resist silencing [37]. In addition, the promoter has an intron for enhancing the expression of the transcript by facilitating transcript splicing [38]. pWPXL also contains a post-transcriptional regulatory element of *woodchuck* hepatitis virus (*WPRE*) that increases transgene expression levels [39]. All the above measures were designed to enable higher expression levels with less silencing of the reprogramming factors, resulting in efficient pluripotency induction. The plasmid also had a loxP site in the 3′ LTR (long terminal repeat) which was copied to the 5′ LTR during the process of reverse transcription of the viral RNA, thereby flanking the reprogramming cassette. The four factors viz. c-Myc, Klf-4, Oct-3/4, Sox2 (MKOS) were stitched into one poly-cistronic construct by 2A self-cleaving peptide sequences [40], [41]. The IRES-HSV-tk cassette was inserted distal to the MKOS cassette. This allowed for the expression of HSV-tk (a negative selection cassette) under the same Ef1-alpha promoter as the MKOS factors. A schematic displaying the various elements is shown in figure 1.

**Figure 2:**
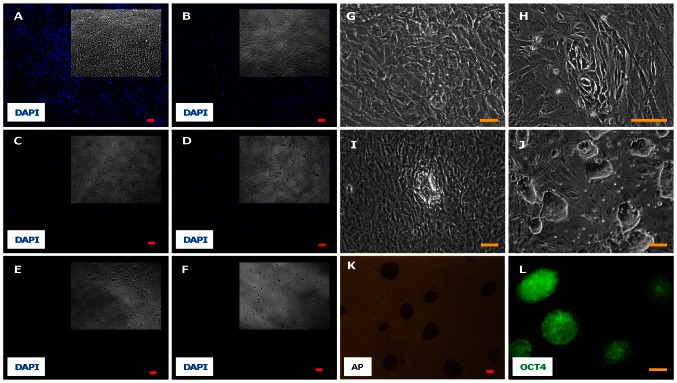
Lentiviral titer estimation and the generation of iPSCs. (A–F) depicts the results of MKOS-tk lentiviral titer estimation. Primary mouse embryonic fibroblasts were exposed to increasing volumes of concentrated lentiviral particles. Subsequently, the cells were selected with ganciclovir for 7 days. The PMEFs (Myh6-GFP+) which took up the lentivirus and were expressing the transgene died due the expression of HSV-tk. contrast images of the wells were taken on the 5^th^ day of ganciclovir selection (inset) and the cells were fixed and stained with DAPI after two more days of selection. The volume of virus applied to 2.5×10^4^ cells were 0 µl (A), 2 µl (B), 10 µl (C), 25 µl (D), 50 µl (E), 100 µl (F). (G–J) shows the evolution of the iPSC colonies from the PMEFs. PMEFs (G) were exposed to the reprogramming lentivirus. 7 days after the transduction small areas of reprogramming arose (H). These areas soon turned into morphologically well-defined colonies (I) which were picked up individually and passaged (J). These colonies stained for the pluripotency marker alkaline phosphatase (K) as well as the embryonic stem cell marker OCT4 (L). Scale bar = 100 µm.

Titration of lentivirus was done following the generation and concentration of the virus. Increasing amounts (2 µl, 10 µl, 25 µl, 50 µl and 100 µl) of lentivirus were applied to 2.5×10^4^ PMEFs for the titration along with a no-virus control. On the 5^th^ day of ganciclovir selection it was observed that 50 µl of virus was killing most of the PMEFs (Figures 2 A–F inset). Doubling the amount of virus to 100 µl improved the selection only marginally. Therefore, we decided against trying out larger amounts of virus and settled on 100 µl for the reprogramming studies. Day 7 DAPI staining confirmed this approach (Figures 2 A–F). PMEFs (Figure 2 G) transduced with 100 ul of virus showed initiation of reprogramming on day 7 (Figure 2 H). By day 12 well-defined colonies had emerged (Figure 2 I). We consistently obtained 100–125 colonies with a starting PMEF population of 25,000. Sixteen individual colonies were picked up on day 18 based on their morphology and passaged on to individual wells after trypsinization (Figure 2 J). The clonal iPSC colonies stained positive for AP (Figure 2 K) and Oct4 (Figure 2 L), suggesting their pluripotent nature. Based on the morphology and staining of the colonies we performed all the subsequent experiments with the two best clones that will be henceforth denoted as clone-1 and clone-2.

**Figure 3:**
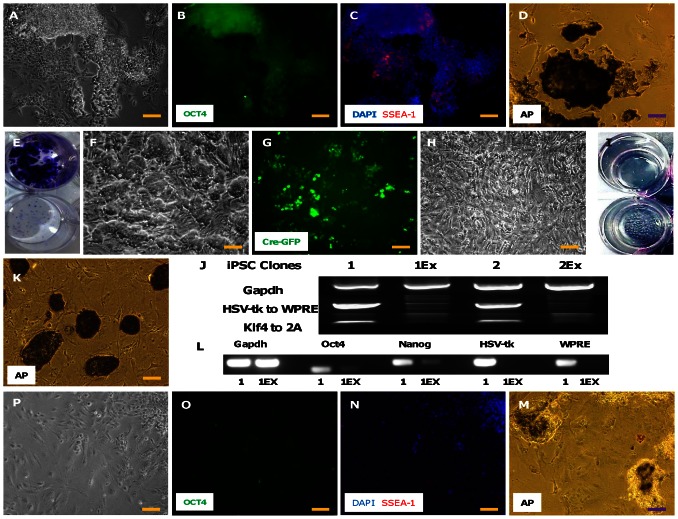
Excision of the reprogramming cassette and its effect on pluripotency markers in differentiating iPSCs. The iPSCs on differentiation displayed many areas consisting of small, rounded cells (A), which stained positive for OCT4 (B), SSEA-1 (C) and alkaline phosphatase (AP) (D). Crystal violet staining of the differentiated iPSCs pre- (E, upper well) and post- (E, lower well) ganciclovir selection showed that the transgenes were not silenced on differentiation, thereby leading to massive cell death in the lower well. Therefore, to stop/decrease the expression of reprogramming transcription factors during differentiation we transfected the iPSC colonies (F) transiently with a CRE:GFP plasmid (G). Massive cellular death ensued after Ganciclovir selection (H)as visualized by the lack of visible colonies in the upper well of figure (I). However, after few days of culture the colonies again started to appear back (lower well of figure I). Genomic PCR confirmed transgene excision in two iPSC clones: pre- (clone #1 and clone #2) and post-excision (clone #1Ex and Clone #2Ex). Genomic Gapdh was used as a control (upper lane) and two areas of the transgene: i) HSV-tk to WPRE (middle lane) ii) Klf4 to 2A (lower lane), were used as the tests. Both the excised clones lacked the test bands. (J). AP staining showed maintenance of pluripotency in the emerging colonies (K). RT-PCR for Oct4 and Nanog in differentiating iPSCs showed a considerable decrease in the expression of both the markers post-excision in both the clones (1 and 2). No HSV-tk and WPRE expression was seen after excision (L). After excision the expression of Oct4, SSEA-1 and Alkaline phosphate were also reduced on ICC in the differentiating cells (M–P). Scale bar = 100 µm.

In a first attempt to apply the cardiac differentiation protocol to the two clones we observed no beating foci or areas displaying green fluorescence (due to activation of the Myh6–GFP transgene) by day 9 of differentiation. To probe whether this lack of differentiation was caused by the continued expression of the reprogramming factors we stained the differentiated cells with OCT4 and SSEA-1 antibodies. We observed several OCT4+ areas (Figure 3 B), which consisted of cells that were rounded and small and had a prominent nucleus (Figure 3 A). Moreover, these areas also stained in a patchy fashion for SSEA-1 (Figure 3 C). On staining with alkaline phosphatase (AP) we observed many regions intensely stained for AP, indicating their lack of differentiation (Figure 3 D). Our assumptions were further bolstered when we observed that almost all the cells died upon the addition of ganciclovir (lower well in Figure 3 E). The differentiated cells in the upper well were not subjected to ganciclovir selection and hence showed abundant crystal violet staining. Consequently we decided to excise out the reprogramming transgenes to decrease the expression of pluripotency factors. It was done by inducing transient expression of Cre recombinase in the form of a Cre:GFP fusion plasmid. Twelve hours after transfection we observed GFP fluorescence in the majority of the colonies, indicating successful expression of Cre recombinase (Figures 3 F,G). Ganciclovir selection was started 5 days after Cre:GFP transfection. This led to massive cell death with obliteration of almost all the visible colonies (Figures 3 H and 3 I upper well). Ganciclovir selection was carried out for 7 days. New colonies which were resistant to ganciclovir soon started to emerge (Figure 3 I lower well). The emerging ganciclovir resistant iPSC colonies were stained with AP to demonstrate the maintenance of pluripotency (Figure 3 K). To prove that ganciclovir resistance of the clones arose from the excision of the transgene and not due to transgene silencing, we performed PCR on genomic DNA to amplify two different areas on the transgene with genomic Gapdh as a control. The ganciclovir-selected clones had successfully removed the transgene as evidenced by the lack of a band even after 40 cycles of amplification (Figure 3 J). RT-PCR showed that the expression levels of Oct4 and Nanog decreased in the day 9 transgene-excised differentiating cells when compared to the unexcised group. No HSV-tk and WPRE expression was observed in the excised clones on RT-PCR (Figure 3 L). SSEA-1, Oct4 and AP activity were all markedly decreased in the excised clones (Figures 3 M–P).

**Figure 4:**
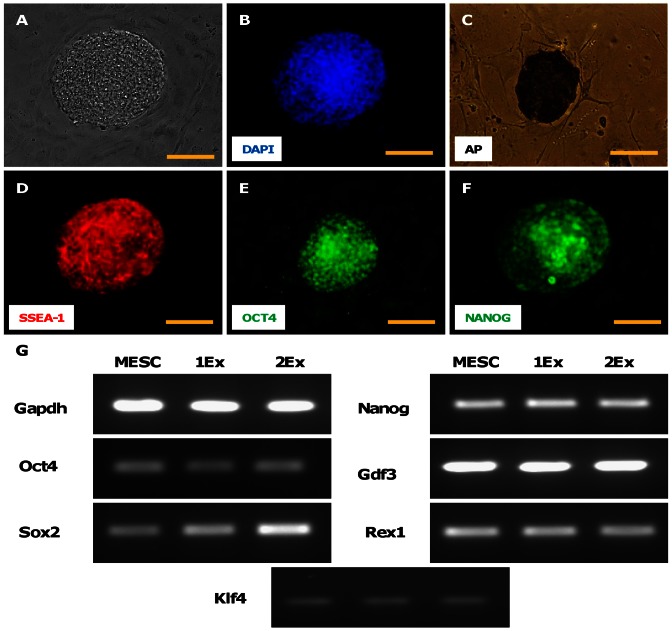
Immunocytochemistry and RT-PCR for stemness and pluripotency markers in transgene-excised iPSCs. The transgene-excised iPSCs were imaged for stemness markers. Phase contrast imaging revealed compact colony morphology (A) with small cells and a prominent nucleus on DAPI staining (B). The colonies stained positive for alkaline phosphatase (C), SSEA-1 (D), OCT4 (E) and NANOG (F). On RT-PCR both the transgene-excised iPSC lines (1Ex and 2Ex) and mouse ES cell line D3 (MESC) expressed stemness markers like Oct4, Sox2, Klf4, Nanog, Gdf3, and Rex1. Gapdh was used as the PCR control (G). Scale bar = 100 µm.

**Figure 5:**
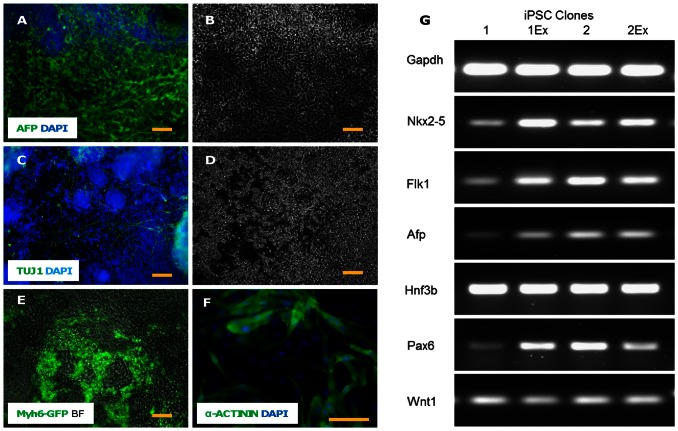
Immunocytochemistry and RT-PCR for differentiation markers in the differentiating transgene-excised iPSCs. The transgene-excised clones were differentiated into three germ layers viz. endoderm, ectoderm and mesoderm. Phase contrast images were taken for cells that we derived by implementing the endoderm differentiation protocol (B) and ectoderm differentiation protocol (D). Merged fluorescent images of endodermal marker: alpha feto protein (AFP) with DAPI (A) and ectodermal marker: TUJ1 (a neuronal marker) with DAPI (C) were acquired. For mesodermal differentiation cardiac differentiation protocol was implemented. Quite a few GFP+ (driven by Myh6 promoter) beating areas were observed (E) which also stained positive for α-ACTININ on ICC (F). Semi-quantitative RT-PCR was also done for differentiation markers: Nkx2.5 and Flk1 for mesoderm, AFP and Hnf3b for endoderm, Pax6 and Wnt1 for ectoderm. Gapdh was used as the PCR control (G). Both the iPSC lines pre- (1, 2) and post-excision (1Ex, 2Ex) were probed. Scale bar = 100 µm.

The excised iPSC clones demonstrated typical ES cell-like compact colony morphology composed of small rounded cells with a large prominent nucleus (Figures 4 A, B). The colonies also stained for embryonic stem cell and pluripotency markers viz. AP, SSEA-1, OCT-4, NANOG (Figures 4 C–F). To further confirm the iPSC status, semi-quantitative RT-PCR was performed with Oct4, Sox2, Klf4, Nanog, Gdf3 and Rex1 primers. Mouse ES D3 line was used as a positive control. Both excised clones were positive for all the markers (Figure 4 G). The clones were then differentiated into ectoderm (neuronal differentiation), endoderm (hepatic differentiation) and mesoderm (cardiac differentiation). The cells stained for Alpha Feto protein (AFP) which is expressed in the hepatocytes during early development (Figures 5 A, B) and Tuj1 which is a neuronal marker (Figures 5 C, D). Robust cardiac differentiation was evidenced by the appearance of multiple green Myh6 GFP+ beating areas (Video S1 & S2) and positive staining with cardiac alpha-ACTININ by the 9^th^ day of differentiation (Figures 5 E, F). On RT-PCR the differentiated cells were positive for the markers of early cardio-vascular mesoderm (Nkx2.5 and Flk-1), endoderm (AFP and Hnf3b) and ectoderm (Pax6 and Wnt1) (Figure 5 G).

**Figure 6:**
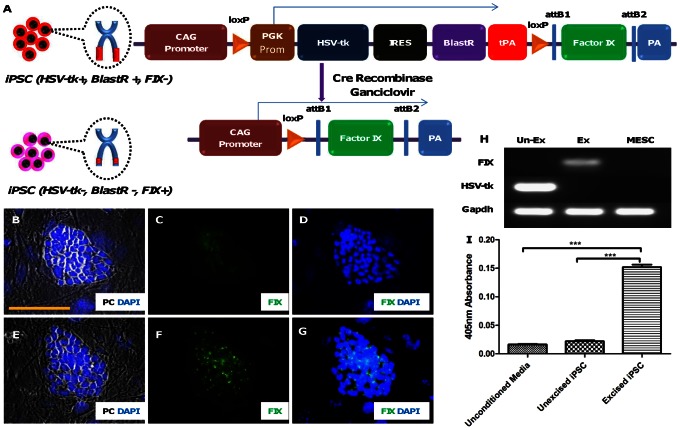
Temporal induction of transgenic FIX expression in iPSCs utilizing the Cre-loxP/HSV-tk/gan selection strategy. Schematic diagram for the vector pCag-lox-PGK-HSV-tk-BlastR-tpA-lox-FIX pre- and post-excision (A). In the unexcised form of the vector HSV-tk and blasticidin resistance protein is expressed from the PGK promoter. On Cre recombinase-mediated excision of the loxP flanked cassette (bottom half of the schematic) the CAG promoter (cytomegalovirus (CMV) enhancer fused to the chicken beta-actin promoter) starts expressing FIX as the tpA stop sequence is lost after the excision event. Both pre- and post-excision iPSC colonies were immunostained with FIX antibody and the nuclear stain DAPI. Phase contrast image of a pre- (B) and post-excision (E) iPSC colony merged with DAPI. Factor IX immunostaining for a pre-excision iPSC colony show only background staining (C) while that of a post-excision iPSC colony show punctate staining (F). FIX staining of a pre-excision (D) and post-excision (G) iPSC colony merged with DAPI staining image. RT-PCR for FIX and HSV-tk was done for pre- and post-excision states (H). HSV-tk was expressed only in unexcised state and FIX was expressed only in excised state. Gapdh was used as the house keeping PCR control. Mouse ES cell D3 line (MESC) was used as the negative control for the experiment. Chromogenic assay to measure the functional activity of the FIX released in the media was done for both the unexcised and excised iPSCs (I). Unconditioned media was used as a negative control. The absorbance of the chromogenic assay product at 405 nm for the media conditioned by excised iPSCs was found to be highly significant (p≤.001, n = 3).

We used the same platform for a proof-of-concept study to insert a therapeutic transgene (human blood coagulation factor IX) in the iPSCs albeit through a non-viral electroporation-based method. Selection of the cells for transgene integration was done with blasticidin antibiotic. Cre recombinase was used to excise the antibiotic cassette followed by HSV-tk/ganciclovir negative selection of the cells. Figure 6 A is a schematic diagram for the vector pCag-lox-PGK-HSV-tk-BlastR-tpA-lox-FIX pre- and post-excision. In the unexcised form, HSV-tk and blasticidin resistance protein are expressed from the PGK promoter. The tpA (triple polyA) STOP signal prevents any leaky expression of FIX before Cre-mediated excision of the selection cassette by acting as a strong transcription termination signal. Factor IX immunostaining (Figures 6 B, C, D), RT-PCR (Figure 6 H) and FIX chromogenic assay (Figure 6 I) of the unexcised transgenic iPSCs were marked by the absence of leaky expression of FIX. Transient expression of Cre recombinase led to the excision of the loxP-flanked selection cassette (HSV-tk-IRES-Blast) as depicted in the bottom half of the schematic (Figure 6 A). The excision event also led to the removal of the tpA signal, thereby triggering CAG promoter-driven expression of the FIX gene. Figures 6 E, F and G show the expression of FIX on ICC which is punctate in appearance and is distributed around the nucleus. This agrees with the fact that inside the cell FIX is concentrated in the endoplasmic reticulum and the Golgi apparatus. On RT-PCR the excised-iPSCs showed expression of FIX with cessation of HSV-tk expression (Figure 6 H). A FIX chromogenic assay showed that the excised-iPSC group produced a higher FIX level compared to the control and the unexcised-iPSC group (one-way Anova; p = 0.001, n = 3) (Figure 6 I). Post hoc comparison using the Tukey's Multiple Comparison Test indicated that the mean value of the excised-iPSC group was significantly (p<0.05) different from the other two groups, but not between the unexcised-iPSC group and the control (Figure 6 I). On an average 33.9 ng of active factor IX was secreted by the excised-iPSCs (seeding density of 50,000 cells/well of a 6-well plate) in 2 ml of media over 24 hours.

## Discussion

In this study we have described the utility of a technology platform (Cre-Lox site-specific transgene excision and subsequent negative selection with HSV-tk/ganciclovir) for iPSC research in two distinct ways.

Initially, the strategy was implemented in a reprogramming construct to ensure better functionality of the iPSCs. A poly-cistronic reprogramming construct (four reprogramming factors stitched together with the self-cleaving 2A factors) was put alongside a HSV-tk negative selection cassette in a lentiviral format. A strong EF1-alpha promoter with an intron along with WPR element was used to increase the reprogramming-transgene expression. We consistently obtained reprogramming efficiencies between 0.4 and 0.5%, which is better than what was reported in the literature with a similar kind of construct and without the addition of reprogramming boosting molecules [42]. The proviral size of the construct was approximately 10.7 kb. Reports of utilizing lentiviral vectors to package and deliver >10 kb of transgenic elements are rare in the literature [43], because of a significant decrease in lentiviral titer with such large transgenes. However, our apprehension was allayed when we were able to maintain a workable titer with our construct. We have also demonstrated the utilization of HSV-tk/ganciclovir negative selection for a simple bright-field-microscopy-based lentiviral titration. This avoids costly and laborious methods for lentiviral vector titration such as ELISA or quantitative RT-PCR. We also demonstrated that reprogramming-transgene excision is necessary as the strong EF1 alpha promoter does not get inactivated in the differentiating iPSC derivatives. This was evidenced by the large Oct4+, SSEA-1+, AP+ areas on staining and also by the expression of HSV-tk and WPRE on RT-PCR of the differentiating cells. Upon administration of ganciclovir to the differentiating derivatives, most of the cells were killed. This indicated that HSV-tk was still being expressed in most of the differentiating cells. We then showed that a transient transfection of the iPSCs with a Cre:GFP plasmid and subsequent selection with ganciclovir led to the emergence of new GFP− colonies which were transgene-free. Massive cell death ensued after ganciclovir selection even though most of the colonies were expressing Cre recombinase. This might be attributed to Cre recombinase expression not translating into transgene excision in most of the cells, and the fact that HSV-tk expression might lead to the death of a neighboring HSV-tk negative cell on exposure to ganciclovir by a phenomenon called “bystander effect” [44]. The iPSCs that emerged after ganciclovir selection maintained their pluripotency as evidenced by the positive staining for pluripotent stem cell markers: AP, Oct4, SSEA-1, Nanog and RT-PCR data. The iPSCs differentiated into all the major lineages and also expressed ectoderm (Pax6, wnt1) endoderm (Hnf3B, AFP) and mesoderm (Flk1 and Nkx2-5) markers on RT-PCR, thereby bolstering the claim that the expression of HSV-tk and subsequent ganciclovir treatment along with the loss of reprogramming transgene had no effect on the maintenance of pluripotency. Moreover the cells that differentiated from the transgene-excised iPSCs showed a decrease in the expression of pluripotency markers. This may account for the appearance of robust Myh6-GFP^+^ beating areas by day-9 of differentiation whereas the differentiating iPSCs from their unexcised counterparts showed delayed beating which appeared on day-11.

We then extended the utility of the technology to generate iPSCs expressing a model therapeutic transgene (human blood coagulation factor IX, FIX). To the best of our knowledge, this is the first work to demonstrate the generation of FIX-expressing iPSCs. We also showed that the non-viral construct can be used to trigger expression of the FIX transgene at a time of our choice (temporal expression control) by transient Cre recombinase transfection, while at the same time removing the antibiotic resistance cassette. This proof-of-concept study with FIX as a model can be translated to any other transgene. Application of gateway technology allows for the easy shuttling of any transgene into the destination vector to generate the working construct. The non-viral nature of the construct may allow for its use in translational studies. Moreover, the construct when placed between two homology arms can be used for targeted transgene insertion. We also showed that in this study the FIX transgene expression was triggered by transient Cre recombinase expression while the cells were still in the pluripotent state. We envisage that the robustness of this construct will also permit the triggering of the target gene in differentiating iPSCs. This ability becomes critical if a tight temporo-spatial control over the transgene expression is desired.

The methods described in this article will not only help in implementing simple, cost-effective and efficient transgenesis in iPSCs but also ensure the subsequent Cre recombinase-mediated transgene removal for full-fledged downstream functionality.

## Supporting Information

Figure S1 **Gateway recombination cloning for generating FIX expression vector.** The gateway destination vector pCag-lox-PGK-HSV-tk-BlastR-tpA-lox-Dest was recombined with the FIX gateway entry vector (pENTRY4-FIX) by LR clonase enzyme to form the final expression vector pCag-loxP-PGK-HSV-tk-BlastR-tpA-loxP-FIX.
(TIF)

Table S1 **RT-PCR primers for iPSCs and their differentiated derivatives.**
(DOCX)

Video S1 **Phase-contrast video microscopy of beating areas.**
(MP4)

Video S2 **Fluorescence video microscopy of Myh6 GFP+ beating areas.**
(MP4)
