# Supplementary figures and images for: A Robust Strategy for Negative Selection of Cre-LoxP Recombination-Based Excision of Transgenes in Induced Pluripotent Stem Cells
Source: PLoS One. 2013 May 22;8(5):e64342. doi: 10.1371/journal.pone.0064342 (PMC3661507; doi:10.1371/journal.pone.0064342)

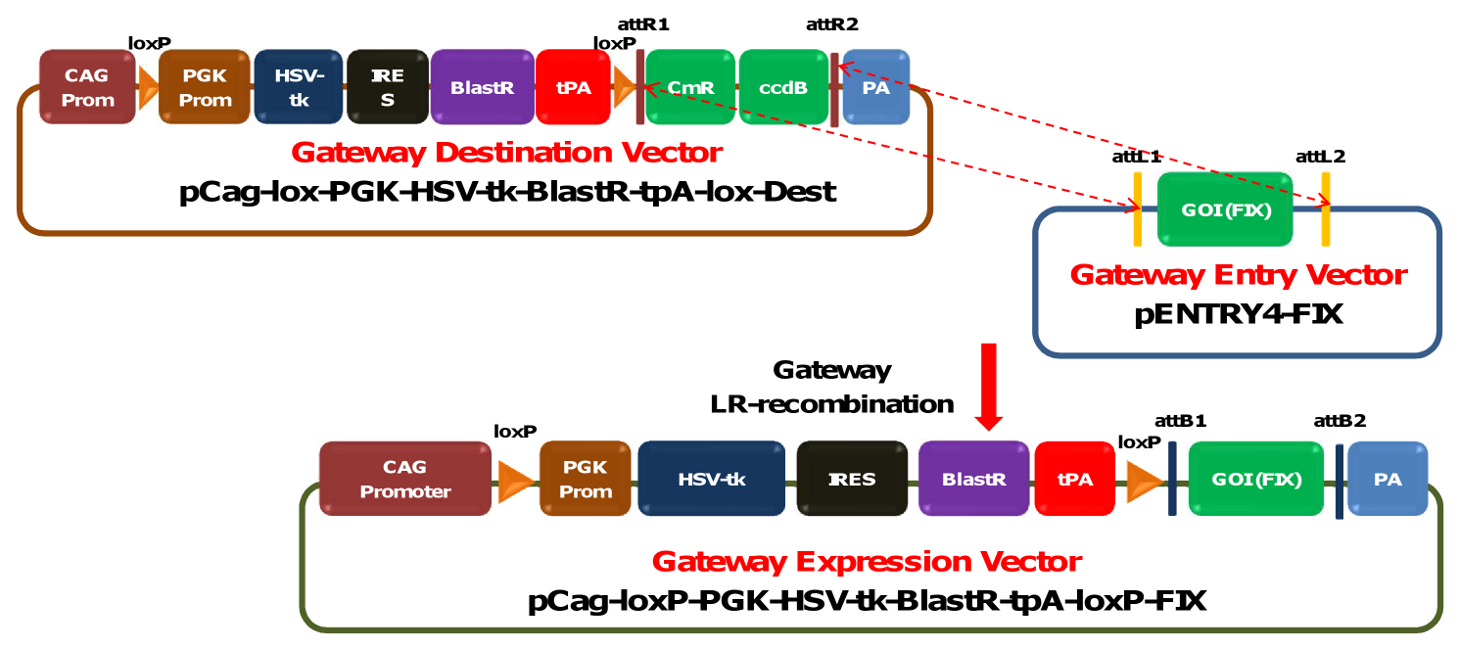

Supplement: Figure S1 — Gateway recombination cloning for generating FIX expression vector. The gateway destination vector pCag-lox-PGK-HSV-tk-BlastR-tpA-lox-Dest was recombined with the FIX gateway entry vector (pENTRY4-FIX) by LR clonase enzyme to form the final expression vector pCag-loxP-PGK-HSV-tk-BlastR-tpA-loxP-FIX. (TIF) [file pone.0064342.s001.tif]
