# Supplementary material for: A Robust Strategy for Negative Selection of Cre-LoxP Recombination-Based Excision of Transgenes in Induced Pluripotent Stem Cells
Source: PLoS One. 2013 May 22;8(5):e64342. doi: 10.1371/journal.pone.0064342 (PMC3661507; doi:10.1371/journal.pone.0064342)
Supplement: Table S1 — RT-PCR primers for iPSCs and their differentiated derivatives. (DOCX) [file pone.0064342.s002.docx]

| **Primer Name** | **Sequence** |
| --- | --- |
| Oct4-F | CCCCATGTCCGCCCGCATAC |
| Oct4-R | TGCTCCTGCCTGGCCCTCAG |
| Sox2-F | GGGGGCAGCGGCGTAAGATG |
| Sox2-R | CCCGCTCGCCATGCTGTTCC |
| Klf4-F | GCCTGCCTCTTCCCCCAGGA |
| Klf4-R | TTGGGCTCCTCTGGCAGGCA |
| Nanog-F | TCGCCCTTCCTCTGAAGAC |
| Nanog-R | TGCTTCTGAAACCTGTCCTTGA |
| Gdf3-F | GGGCCTCGCAGGACTTATG |
| Gdf3-R | TGGTCGCAGGTTATAGTAGGAC |
| Rex1-F | AAGCTGCCAGCCAGTAACC |
| Rex1-R | ACCAACTTTCCCGATGACATCT |
| Gapdh-F | TGCGACTTCAACAGCAACTC |
| Gapdh-R | CTTGCTCAGTGTCCTTGCTG |
| WPRE-F | ACTGTGTTTGCTGACGCAAC |
| WPRE-R | CAACACCACGGAATTGTCAG |
| HSV-tk-F | TACCCGAGCCGATGACTTAC |
| HSV-tk-R | CCGATATGAGGAGCCAGAAC |
| Hnf3b-F | TGGTCACTGGGGACAAGGGAA |
| Hnf3b-R | GCAACAACAGCAATAGAGAAC |
| Afp-F | TCGTATTCCAACAGGAGG |
| Afp-R | AGGCTTTTGCTTCACCAG |
| Pax6-F | CCATCTTTGCTTGGGAAATCCG |
| Pax6-R | GCTTCATCCGAGTCTTCTCCGTTAG |
| Wnt1-F | TCCTCCACGAACCTGTTGACGG |
| Wnt1-R | GATTGCGAAGATGAACGCTGTTTC |
| Flk1-F | TTTGGTTTTGGAAGGTTTGC |
| FLK1-R | GATGAGGAAGGAGCAAGCTG |
| Nkx2.5-F | AACAGCAACTTCGTGAACTTTGG |
| Nkx2.5-R | GAGTCATCGCCCTTCTCCTAAA |
